# Graduates of Medical Colleges

**Published:** 1856-08

**Authors:** 


### EDITORIAL.

Our readers will require of us no apology for occupying so much space in this number of the *Journal* with the Report on Practical Medicine, read at the last meeting of the State Medical Society, by Dr. Samuel Thompson, of Albion. It is a document containing many valuable suggestions, not only of a practical nature, in reference to the prevalence and treatment of disease in the middle and southern portions of this State, but also in reference to modes of investigation, records of cases, &c. The remainder of the Report, together with the Annual Address of the retiring President of the Society, will be *contained* in our next issue. If these papers should cause some delay in the publication of Communications from our correspondents, they must excuse us, for their favors shall be attended to as early as practicable.

---

*Graduates of Medical Colleges.*

The whole number of graduates from the medical colleges of the United States, for the year 1856, is about 1,450. The following 18 schools stand highest on the list in the number of their graduates, viz.:

| | |
|---|---|
| Jefferson Medical College | 215 |
| University of Pennsylvania | 140 |
| University of New York | 98 |
| University of Nashville | 85 |
| Medical College of South Carolina | 85 |
| Medical College of Georgia | 73 |
| University of Louisiana | 65 |
| University of Louisville | 61 |
| St. Louis Medical College | 50 |
| Rush Medical College | 42 |
| College of Physicians and Surgeons | 40 |
| Cleveland Medical College | 38 |
| Pensylvania Medical College | 37 |
| New York Medical College | 35 |
| University of Michigan | 30 |
| University of Missouri | 28 |
| Massachusetts Medical College | 28 |
| Philadelphia College of Medicine | 21 |

